# Tracheostomy in children: a ten-year experience from a tertiary center in southern Brazil^☆^^☆☆^

**DOI:** 10.1016/j.bjorl.2016.08.002

**Published:** 2016-08-24

**Authors:** Cláudia Schweiger, Denise Manica, Carolina Fischer Becker, Larissa Santos Perez Abreu, Michelle Manzini, Leo Sekine, Gabriel Kuhl

**Affiliations:** a Hospital de Clínicas de Porto Alegre, Serviço de Otorrinolaringologia, Porto Alegre, RS, Brazil; b Universidade Federal do Rio Grande do Sul (UFRGS), Programa de Pós-graduação em Epidemiologia, Porto Alegre, RS, Brazil; c Universidade Federal do Rio Grande do Sul (UFRGS), Departamento de Oftalmologia e Otorrinolaringologia, Porto Alegre, RS, Brazil

**Keywords:** Tracheostomy, Child, Epidemiology, Traqueostomia, Criança, Epidemiologia

## Abstract

**Introduction:**

Children may require tracheostomy due to many different health conditions. Over the last 40 years, indications of tracheostomy have endorsed substantial modifications.

**Objective:**

To evaluate pediatric patients warranted tracheostomy at our Hospital, in regard to their indications, associated comorbidities, complications and decannulation rates.

**Methods:**

Retrospective study concerning patients under 18 years of age undergoing tracheostomy in a tertiary health care center, from January 2006 to November 2015.

**Results:**

123 children required a tracheostomy after ENT evaluation during the study period. A proportion of 63% was male, and 56% was under one year of age. Glossoptosis was the most common indication (30%), followed by subglottic stenosis (16%) and pharyngomalacia (11%). The mortality rate was 31%. By the end of this review, 35 children (28.4%) had been decannulated, and the fewer the number of comorbidities, the greater the decannulation rate (0.77 ± 0.84 vs. 1.7 ± 1.00 comorbidities; *p* < 0.001).

**Conclusion:**

Tracheostomy in children is a relatively frequent procedure at our hospital. The most common indications are glossoptosis and subglottic stenosis. A high mortality rate was found, potentially substantiated by the high number of critical care patients with chronic neurological conditions in this cohort. Our decannulation rate is slightly below other series, probably because of the greater amount of patients with comorbidities.

## Resumo

**Introdução:**

As crianças podem necessitar de traqueostomia devido a diferentes problemas de saúde. Ao longo dos últimos 40 anos, as indicações de traqueostomia passaram por mudanças substanciais.

**Objetivo:**

Avaliar pacientes pediátricos com traqueostomia no nosso Hospital, no que diz respeito às suas indicações, comorbidades associadas, complicações e taxas de decanulação.

**Método:**

Estudo retrospectivo de pacientes com menos de 18 anos de idade submetidos a traqueostomia em um centro de saúde terciário, de janeiro de 2006 a novembro de 2015.

**Resultados:**

123 crianças precisaram de uma traqueostomia após avaliação otorrinolaringológica durante o período do estudo. Do total, 63% era do sexo masculino e 56% menores de um ano. Glossoptose foi a indicação mais comum (30%), seguida por estenose subglótica (16%) e faringomalácia (11%). A taxa de mortalidade foi de 31%. Até o fim deste artigo, 35 crianças (28,4%) haviam sido decanuladas e quanto menor o número de comorbidades, maior foi a taxa de decanulação (0,77 ± 0,84 vs. 1,7 ± 1,00 comorbidades; *p* < 0,001).

**Conclusão:**

A traqueostomia em crianças é um procedimento relativamente frequente em nosso hospital. As indicações mais comuns são glossoptose e estenose subglótica. Uma alta taxa de mortalidade foi encontrada, potencialmente comprovada pelo elevado número de pacientes críticos com condições neurológicas crônicas nessa coorte. Nossa taxa de decanulação está ligeiramente abaixo de outras séries, provavelmente por causa da maior quantidade de pacientes com comorbidades.

## Introduction

Tracheostomy is one of the oldest and most commonly performed surgical procedures among critically ill patients. Children require tracheostomy for many different reasons, and those with a chronic tracheostomy constitute an important subgroup of children who are at risk for airway compromise.

Although a life saving surgery, tracheostomy in children is more challenging when compared to those performed in adults, and it is associated with higher rates of morbidity and mortality. Overmore, the risk of complications increases with age among pediatric patients.^1^, ^2^

Over the last 40 years, there has been a transition in tracheostomy indications. During 1970s, the main indication of tracheostomy was upper airway obstruction secondary to an acute inflammatory disease such as epiglottitis, croup or laringotracheitis.^2^ With the introduction of vaccines against *Haemophilus influenzae* and *Corynebacterium diphteriae* and the adoption of routine use of endotracheal intubation as an alternative to tracheostomy, there was a decline in such indications.

On the other hand, the improvement of support care and therapeutics in premature infants and children with congenital anomalies increased the survival rates of these children, which often required protracted periods of endotracheal intubation and mechanical ventilation. Children with endotracheal intubation may need mechanical ventilation for conditions such as pulmonary and cardiac malformations, chronic pulmonary insufficiency, neurological disorders and cervical trauma. In the other hand, craniofacial malformations and anatomical/functional changes of the larynx, such as subglottic stenosis, tracheomalacia and tumors, are among the obstructive causes.^3^

A recent study from a Scotland tertiary center^3^ found long-term ventilation as the main indication for tracheostomy in their series of 111 children and cited some articles from United Kingdom hospitals with similar findings. However, a study in New Zealand^4^ included 122 patients undergoing tracheostomies between 1987 and 2003 and found that the obstruction of upper airway was the indication in 70% of the patients. More recently, an American study published in March, 2013,^5^ which reviewed data from 158 patients, raised the hypothesis that indications of tracheotomy would be changing again, with a reduction of indications for prolonged endotracheal intubation and an increase in the group of craniofacial anomalies and upper airway obstruction.

Thus, our objective is to evaluate children who warranted tracheostomy at our Hospital, in regard to their indications, associated comorbidities, complications and decannulation rates.

## Methods

This is a retrospective study of all tracheostomies performed in children by the ENT Unit of our Hospital between January 2006 and November 2015. Our Hospital is a tertiary referral center at Southern Brazil, which receives high complexity patients such as pediatric airway disorders. Patients’ files were reviewed with respect to the following variables: demographics, comorbidities, indication for procedure, complications related to the tracheostomy and decannulation.

Surgeries were performed by resident doctors under consultant supervision. All patients were under general anesthesia during procedure and were admitted to the pediatric intensive care unit after surgery. A standard procedure technique was applied, with horizontal skin incision and a vertical incision in the trachea. Two parallel sutures were placed on each side of the tracheal incision. The sutures in the trachea were removed one week later.

The protocol of the study was approved by the Research and Ethics Committee of our Hospital (n° 14-0650).

Data are shown in median (interquartile range) or mean ± Standard Deviation. Mann–Whitney *U* test was used for statistical analysis when appropriate.

## Results

### Patient demographics

**Figure 1:**
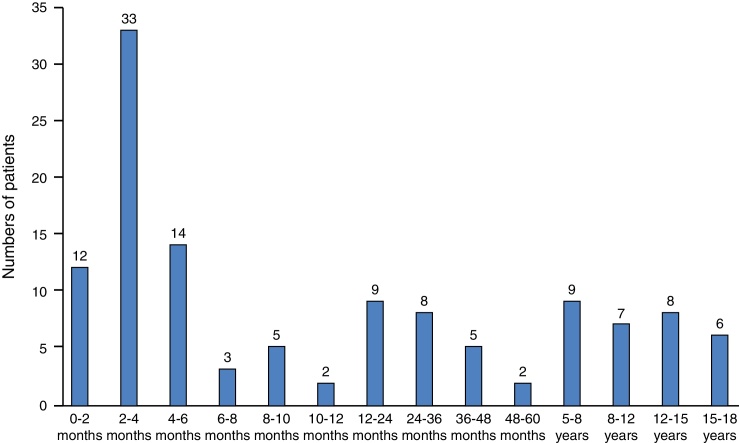
Distribution of patients in regard to their age.

Between January 2006 and November 2015, 146 children evaluated by the ENT Unit required a tracheostomy. Data was available from 123 patients. Seventy-eight (63%) patients were male. Almost half of them had their tracheostomy performed under six months of age (48%) and 69 patients (56%) were under one year of age at the time of the procedure. The median age of tracheostomy was 7 months, with age ranging from 8 days to 17 years (Fig. 1).

**Figure 2:**
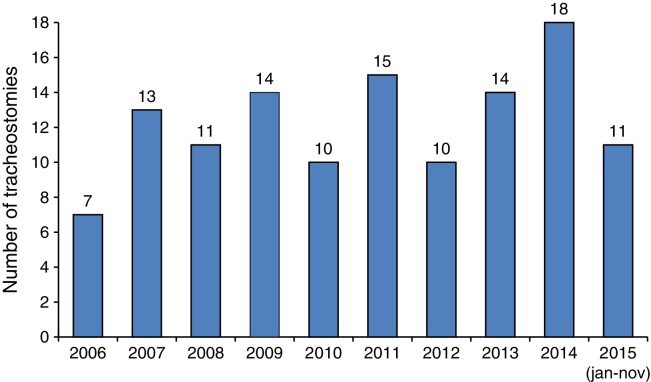
Number of tracheostomies performed per year.

A median of 12 (interquartile range: 10–14.25) tracheostomies was performed each year. The number of procedures per year remained stable over the study period (Fig. 2).

### Indications

Glossoptosis was the most common indication (30%), followed by subglottic stenosis (16%) and pharyngomalacia (11%). Other causes included laryngomalacia, prolonged intubation, acute lesions of the larynx leading to extubation failure, vocal fold palsy and laryngeal papillomatosis (Table 1). Some children had more than one indication for tracheostomy.

**Table 1:** Indications for tracheostomy.

Indications	N° of patients (%)
Glossoptosis	37 (30)
Subglottic stenosis	20 (16)
Pharyngomalacia	14 (11)
Prolonged ventilation	13 (10)
Laryngomalacia	13 (10)
Acute lesions of the larynx leading to extubation failure	10 (8)
Vocal fold palsy	5 (4)
Intubation failure	5 (4)
Laryngeal papillomatosis	4 (3)
Glottic stenosis	4 (3)
Maxillary hipoplasia	4 (3)
Linfangioma	2 (1)
Mucopolissacaridosis	1 (0.8)
Hemangioma	1 (0.8)
Laryngeal cleft	1 (0.8)
Laryngitis	1 (0.8)
Pulmonary toilet	1 (0.8)

Upper airway obstruction was the indication for tracheostomy in 102 patients (83%), concerning disorders like glossoptosis, subglottic and glottic stenosis, pharyngomalacia, laryngomalacia, vocal fold palsy, acute lesions of the larynx leading to extubation failure (post-intubation laryngitis), craniofacial abnormalities and airway tumors. Pulmonary toilet, intubation failure and long-term ventilation were non-obstructive causes for tracheostomy.

Twenty-one patients (17%) had their tracheostomy placed after surgical failure. Supraglottoplasty was not successful in five patients, and osteomandibular distraction as well as resection of papillomatosis lesions could not revert the obstruction in four patients each one (Table 2).

**Table 2:** Tracheostomy after surgical failure.

Surgical procedure	Number of patients (%)
Supraglotoplasty	5 (4)
Osteomandibular distraction	4 (3)
Excision of papillomatosis	4 (3)
Balloon dilatation for SGS	4 (3)
Laryngotracheoplasty	1 (0.8)
Uvulopalatoplasty	1 (0.8)
Laser procedure for SGS	1 (0.8)
Treatment of airway acute lesions	1 (0.8)

### Comorbidities

The majority of children (84%) had comorbidities. Fifty-five children had at least one comorbidity, corresponding to 45% of this series, and approximately 40% of children had two or more comorbidities. Only 20 patients had no parallel health conditions.

Nearly 41% presented neurological conditions (cerebral palsy, encephalopathy, epilepsy, neuromuscular disorders) and 27 patients had some syndromic disorder that included Treacher-Collins syndrome and Down syndrome. The main comorbidities presented by the 103 children in the series are listed on Table 3. Besides neurological and syndromic conditions, comorbidities also included prematurity, cardiac congenital disease and lung disease.

**Table 3:** Comorbidities found in this series.

Comorbidities	Number of patients (%)
Epilepsy	30 (24)
Cerebral palsy	26 (21)
Prematurity	16 (13)
Pneumopathy	16 (13)
Encephalopathy	13 (11)
Robin Sequence	12 (10)
Congenital cardiac disease	9 (7)
Chromosomal changes	8 (6)
West syndrome	5 (4)
Neuromuscular disorder	4 (3)
Hydrocephalus	4 (3)

### Complications

Complications were divided into perioperative, and early and late postoperative (Table 4). Perioperative complications were defined as those occurring during the surgical procedure or immediately after. Those occurring during the first postoperative week were considered early complications, and late complications were defined as those occurring after the first week.

**Table 4:** Complications.

Complications	N° of patients (%)^a^
Perioperative	10 (8)
Pneumothorax	6
Subcutaneous emphysema	3
Pneumomediastinum	2
Death	1
Early postoperative	7 (6)
False tract of the cannula	4
Accidental decannulation	3
Wound infection	2
Late postoperative	38 (31)
Stomal and/or supra-stomal granulomas	23
Accidental decannulation	7
Cannula obstruction	5
Papillomatosis extension to the trachea	2
Tracheal stenosis	2

a Percentage of all tracheostomized children.

Perioperative complications occurred in ten children (8.1%) and included six cases of pneumothorax, three patients with subcutaneous emphysema and two children with pneumomediastium. There was no episode of significant bleeding. One death occurred during the procedure, in a child with Otopalatodigital syndrome. The cause of death was impossibility of ventilation due to pulmonary problems.

Early postoperative bleeding, accidental decannulation, wound infection and false tract of the cannula occurred in seven patients.

Late postoperative complications occurred in 38 patients (31%). Twenty-three patients presented with stomal and/or supra-stomal granulomas adjacent to the tracheostomy. Some of the granulomas were excised and others were treated with chemical ablation. Cannula obstruction by plug of secretion, late accidental decannulation, papillomatosis extension to the trachea and tracheal stenosis also occurred.

### Length of tracheostomy stay and decannulation

Time between tracheostomy and decannulation ranged from less than one month to 7 years (median of 5 months). Six patients required surgical closure of the tracheostomy. By the end of this review, 35 children from this cohort had been decannulated (28%).

The decannulation protocol of our institution includes cannula downsizing and its occlusion after specific treatment of the airway pathology. A laryngoscopy is always performed prior to the cannula downsizing, in order to confirm the patency of the airway.

The majority of decannulated children had no comorbidities or only one comorbidity, corresponding to 86% of cases. Subglottic stenosis was the main indication of tracheostomy in the decannulated children (15 patients).

**Figure 3:**
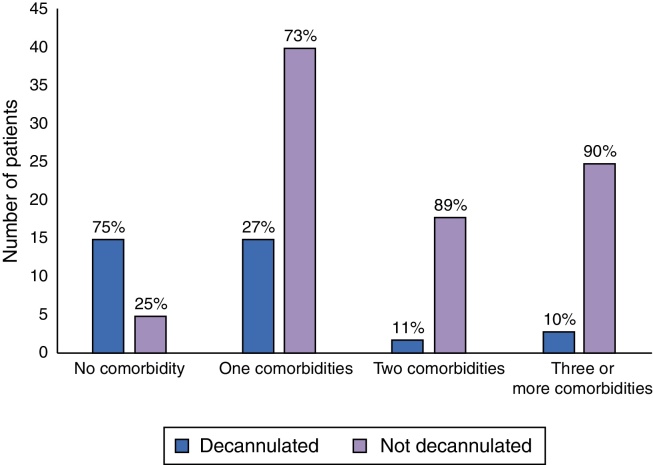
Association between the number of comorbidities and decannulation rates.

Analyzing the association between comorbidities and decannulation rates, one can notice that the fewer the number of comorbidities, the greater the decannulation rates (0.77 ± 0.84 comorbidities vs. 1.7 ± 1.00 comorbidities in decannulated vs. non-decannulated patients, respectively; *p* < 0.001). Data are shown in Fig. 3.

### Mortality

Thirty-eight (31%) children died during study period, and sepsis was the cause in fourteen. Acute respiratory insufficiency caused the death in eight children; pneumonia and clinical complications in another 11 patients. There were apparently no deaths caused by accidental decannulation or cannula obstruction. However, we were unable to identify a death cause in six children.

## Discussion

In comparison with adult population, where indications seem to remain the same over the years, studies recently have shown a historical transition in indications for tracheostomy in children. In developed countries, acute epiglottitis and laryngotracheobronchitis no longer represent an indication for tracheostomy, due to the use of the endotracheal intubation in these cases, besides *Haemophilus influenza* type B vaccine for epiglottitis.^3^ In this cohort, only one child still had a tracheostomy for acute inflammation of the upper airway (lupus laryngitis).

The commonest indications for pediatric tracheostomy are prolonged ventilation studies and obstruction of the upper airway. In a recent study with 111 children published by a Scotland tertiary center,^3^ 32% of patients had their tracheostomy indicated for long-term ventilation, followed by craniofacial abnormality causing airway obstruction in 18% of children and subglottic stenosis in 14%. Their numbers are similar to those of United Kingdom hospitals.^6^, ^7^ Douglas et al. attributed this to the elevated number of patients with significant medically problems surviving long term, as a result of the improvement in neonatal intensive care.

In our series of patients, the most common indication of tracheostomy was upper airway obstruction, caused by craniofacial abnormalities and subglottic stenosis. The majority of the obstructive causes were glossoptosis, with Robin Sequence figuring in first place among our patients.

Owzen et al.^1^ from Turkey and Mahadevan et al.^4^ from New Zealand also reported that airway obstruction accounted for the majority of indications of tracheostomy. In Owzen's series of 282 patients, airway obstruction made up 72% of indications. Mahadevan presented a larger series, and also showed that 70% of the tracheostomies occurred because of airway obstruction.

Syndromic patients represented a large number of our tracheostomized children. The obstruction usually found in these conditions can be associated to craniofacial abnormalities leading to anatomical or functional obstructions, as well as a hypotonic status. Anatomical obstructions can be seen on imaging examination or rigid laryngotracheal endoscopic evaluation, but functional disorders are better evaluated by flexible endoscopy of the airway. In our hospital, all pediatric patients with airway obstruction suspicion are evaluated by rigid and flexible airway endoscopy, which allow us to diagnose conditions as glossoptosis, pharyngomalacia and tracheomalacia.

Our hospital is a reference center in the treatment of Pierre Robin Syndrome patients, and that could explain the increased number of glossoptosis found in our population.

Regarding the 16% of subglottic stenosis as indication for tracheostomy, we have been witnessing a change on recent years, after the implementation of balloon dilation for subglottic acute lesions. Recently, the great majority of children evaluated because of acute subglottic obstructive lesions are submitted to endoscopic balloon dilation, with excellent results and avoidance of tracheostomy. Since 2009, with the introduction of balloon dilation, only four tracheostomies were performed because of subglottic stenosis.

Thirteen patients were tracheostomized because of laryngomalacia. All of them had comorbidities and 85% had neurological disorders. Besides laryngomalacia, all patients had parallel indications for tracheostomy, like glossoptosis and pharyngomalacia.

Four patients required tracheostomy for obstructive recurrent papillomatosis lesions even after cidofovir and bevacizumab therapies, and two presented with tracheal extension as a complication of the tracheostomy. This reality is supposed to change after the implementation of the HPV vaccine and continuous education on prenatal care.

A mean of 13.6 tracheostomies was performed each year. In our series, the mean number of procedures remained stable over the years, unlike other studies, that have reported a decrease in the number of pediatric tracheostomies performed.^3^, ^4^, ^5^, ^6^

Analysis of the demographic characteristics of patients in the literature revealed that the majority of tracheostomies were performed in children under one year of age.^2^, ^8^, ^9^ Our series showed the same trend. This could be probably explained by the increasing survival rates among premature and syndromic patients and the higher severity of obstructive problems at this age.^10^

The tracheostomy-related complications rates in the literature are around 40%, with numbers ranging from 18% to 56%^3^, ^5^, ^11^, ^12^ The most common complications are pneumothorax, pneumomediastium, stomal and supra-stomal granulomas, cannula obstruction, false tract and accidental decannulation.

In our series, perioperative and early postoperative complications rates were low, but still significant (8% and 6%, respectively). These rates show that the tracheostomy is not a procedure to be underestimated about its risks and complications.

Pneumothorax was the most common perioperative complication, and false tract was the most common early postoperative complication. On the other hand, late postoperative complications were higher than reported in literature, with the development of stomal and/or supra-stomal granulomas in 23 children. It could probably be explained by the variety of cannula brands used. Besides, some complications are secondary to poor education on tracheostomy care and low socio-economical conditions, leading to accidental decannulations and cannula obstruction.

Decannulation is always the objective of surgeons and families, but this is not always possible. Studies show that the decannulation rates vary around 35–75%,^1^, ^2^, ^3^, ^4^, ^5^, ^6^ and the highest rates are found in studies with higher prevalence of airway obstruction warranted tracheostomies. Studies with lower rates of decannulation are usually those with a higher number of neurologically compromised patients, as in our series.

Of our decannulated patients, 41% had subglottic stenosis and 44% had no comorbidities. This elevated rate of decannulation in children with subglottic stenosis can be explained by the use of balloon dilations in acute subglottic lesions and successful open technique surgeries in the recent years. Only 16% of the tracheostomized patients were otherwise healthy (no comorbidities or craniofacial malformations) and most of them were decannulated. Robin Sequence, with obstructive glossoptosis and neurological disorder patients represent a relevant portion of still non-decannulated patients.

The mortality rate of tracheostomized patients is relatively high, between 14 and 19%. In our series, it represented 32% of the tracheostomized children. However, most of these deaths were related to underlying comorbidities of these patients and not directly to the presence of the tracheostomy.^2^, ^4^, ^11^, ^13^, ^14^ The elevated number of critical patients with chronic neurological conditions can also explain our increased mortality rate. There were some deaths at home with no identifiable cause.

## Conclusion

Tracheostomy in children is a relatively frequent procedure at our hospital. The commonest indications are glossoptosis and subglottic stenosis. Our decannulation rate is slightly below other series, probably because of the increased amount of patients with comorbidities. The perioperative complication rate is low compared to the literature.

## Conflicts of interest

The authors declare no conflicts of interest.
